# Innate signaling within the central nervous system recruits protective neutrophils

**DOI:** 10.1186/s40478-019-0876-2

**Published:** 2020-01-08

**Authors:** Reza Khorooshi, Joanna Marczynska, Ruthe Storgaard Dieu, Vian Wais, Christian Rønn Hansen, Stephanie Kavan, Mads Thomassen, Mark Burton, Torben Kruse, Gill A. Webster, Trevor Owens

**Affiliations:** 1 Department of Neurobiology Research, Institute of Molecular Medicine, University of Southern Denmark, J.B. Winsloewsvej 25, DK-5000 Odense C, Denmark; 2 Laboratory of Radiation Physics, Odense University Hospital, Odense, Denmark; 3 Institute of Clinical Research, Department of Clinical Genetics, University of Southern Denmark, Odense University Hospital, Odense, Denmark; 4 Innate Immunotherapeutics, Auckland, New Zealand

**Keywords:** Innate signaling, Phagocytosis, Neutrophils, EAE, CNS homeostasis

## Abstract

There is great interest in understanding how the central nervous system (CNS) communicates with the immune system for recruitment of protective responses. Infiltrating phagocytic monocytes and granulocytes are implicated in neuroinflammation in multiple sclerosis and its animal model experimental autoimmune encephalomyelitis (EAE). To investigate how CNS endogenous signals can be harnessed to promote anti-inflammatory programs, we have used a particulate Toll-like receptor 9 and nucleotide-oligomerization domain 2 bispecific innate ligand (MIS416), to address whether its phagocytosis within the CNS recruits protective myeloid cells. We find that MIS416 injected intrathecally into the cerebrospinal fluid via the cisterna magna induced a local chemokine response that recruited blood-derived monocytes and neutrophils to the CNS. These cells phagocytosed MIS416. The increase in EAE severity normally seen from time of onset did not occur in mice receiving MIS416. This suppression of disease symptoms was dependent on expression of the type I interferon receptor (IFNAR). Transfer of intrathecal MIS416-induced neutrophils suppressed EAE in recipient mice, while monocytes did not transfer protection. MIS416-induced neutrophils showed increased IL-10 expression that was IFNAR1-driven. In contrast to intrathecal administration, intravenous administration of MIS416 led to monocyte but not neutrophil infiltration to the CNS. We thus identify a CNS-intrinsic and -specific phagocytosis-induced recruitment of anti-inflammatory neutrophils that contribute to CNS homeostasis and may have therapeutic potential.

## Introduction

Interaction between the central nervous system (CNS) and the immune system exists for benefit to the host. Nevertheless neuroinflammation is considered deleterious to brain homeostasis and normal functioning, as evidenced by clinical pathologies such as Multiple sclerosis and its animal model Experimental Autoimmune Encephalomyelitis (EAE). The CNS is relatively isolated from the immune system as well as to cellular traffic due to a more complex barrier compared to other organs. This poses questions as to how cells of the immune system can effectively access the CNS that they should protect. Immune effector cells are directed to sites of infection or damage by signals emanating from the affected tissue, which include cytokines and chemokines, and CNS-immune interaction can be assumed to follow similar guiding principles. This focuses attention to cell sources of chemokines within the CNS. There is a need to better understand how the CNS communicates with the immune system for recruitment of protective responses.

The CNS contains multiple subpopulations of resident myeloid cells, which play a critical role in induction and promotion of immune responses. Microglia are CNS-resident myeloid cells that fulfil classical tissue macrophage roles within the CNS parenchyma [20]. Extraparenchymal myeloid cells, that are in direct contact with cerebrospinal fluid (CSF), include perivascular and meningeal macrophages [19]. They share prenatal origin with microglia, as well as major elements of their transcriptional profile, and like microglia, these CSF cells are not normally replaced by blood-derived cells [19]. They are implicated functionally in host response to pathogens, which implies an immune recruitment capacity. Extraparenchymal myeloid cells can be distinguished from microglia not only by their location but also by their higher levels of cell surface expression of the tyrosine phosphatase CD45, which they share with blood-derived myeloid cells and lymphocytes [19].

Peripheral myeloid cells, in tissues and in blood, share host-protective pathogen response capacity with CNS-resident cells but differ in their high turnover, short half-life and in their transcriptional profile [19]. Infiltration to tissues from blood by monocyte-derived macrophages and by granulocytes is associated with inflammatory pathology, which is often deleterious, especially in an autoimmune context. Nevertheless, cells of these lineages can be directed towards anti-inflammatory and tissue protective roles, and there is much interest in whether and how suppressive monocytes and granulocytes, especially neutrophils, can be induced and directed [3].

Innate signaling receptors are implicated in primary response to pathogens as well as to tissue damage. Such receptors include members of the Toll-like receptor (TLR) and nucleotide-oligomerization domain (NOD)-containing protein families. These pattern recognition receptors are implicated in recruitment and mobilization of immune effectors both in protective innate responses as well as in induction of adaptive immune responses [9]. Many of these receptors are located intracellularly, such as TLR9 in phagosomes and endosomes, or NOD2 in the cytosol, as befits their role in cells that phagocytose microbes and damaged cells. CNS-resident myeloid and glial cells as well as neurons express varied and individual profiles of innate receptors [24]. We have shown TLR3 and its ligand Poly-I:C to play protective roles in antiviral and autoimmune inflammatory responses within the CNS [11, 22].

Here we have used a bispecific innate-signaling immunomodulatory microparticle, in order to probe the ability of phagocytic myeloid cells within the CNS to recruit a protective myeloid response. Microparticle Immune Stimulator-416 (MIS416), originally developed as a vaccine adjuvant [5], is a modified bacterial cell body that is a specific agonist for TLR9 and NOD2 [16, 34]. MIS416 has been shown to suppress EAE when given peripherally to mice [34], and it has also been shown to enhance protection in mesenchymal stem cell therapy and to target myeloid suppressor cells in tumors [10, 14]. We reasoned that direct delivery of MIS416 into the CSF should trigger protective myeloid responses within the CNS. Our findings show that intrathecal administration of MIS416 induced CNS-specific myeloid cell chemokine-driven recruitment of neutrophils from the blood that suppressed EAE in a Type I-interferon-dependent manner.

## Materials and methods

### Mice

Female C57BL/6j bom mice (abbr B6) aged 6–8 weeks were obtained from Taconic Europe A/S (Lille Skensved, Denmark) and maintained in the Biomedical Laboratory, University of Southern Denmark. Albino (C57BL/6-Tyr^c-2J^) IFNβ^+/Δβ-luc^ mice (IFNβ/luciferase reporter mice), Interferon-α receptor 1 – deficient (IFNAR1-KO) mice (C57BL/6 background) and Yellow Fluorescent Protein (YFP) (IFN-β^mob/mob^) IFNβ knock-in mice were bred and housed in the Biomedical Laboratory, University of Southern Denmark. All experiments were conducted in accordance with the Danish national ethical committee (Animal Experiments Inspectorate (approval number 2014-15-0201-00369)).

### Construction of bone-marrow chimeric mice

To identify and track leukocytes trafficking to the CNS, head shielded female B6 mice were lethally irradiated by 10 Gy in one fraction with opposing 6 MV beams, with 10 mm of bolus on both sides of the mice. The dose was simulated and validated using pilot mice, which were CT-scanned in the treatment setup. Within 24 h post irradiation mice received 10–15 × 10^6^ of bone marrow cells isolated from Ly5.1 donor mouse [17].

Recipient mice were fed high nutrient fodder supplemented with DietGel Recovery and probiotic paste ZooLac for a period of 7 days. Additionally, drinking water was supplemented with antibiotic Oxytetracyclin (400 mg/l) and later replaced by acidic water (pH 3). Chimerism level was verified by flow cytometric analysis of blood samples collected from recipient mice 8 weeks post bone marrow transplantation.

### EAE induction

B6 and IFNAR-KO mice were immunized by subcutaneous (sc) injection in the flanks of an emulsion containing myelin oligodendrocyte glycoprotein (MOG) p35–55 (100 μg) and complete Freund’s adjuvant with heat-inactivated *Mycobacterium tuberculosis* (200 μg; Difco Laboratories). At the time of immunization and 1 day post immunization the animals received an intraperitoneal (ip) injection of *Bordetella pertussis* toxin (0.3 μg, Sigma- Aldrich). Animals were then monitored for loss of body weight and EAE symptoms. The EAE grades were defined as follows: grade 0, no signs of disease; grade 1, weak or hooked tail; grade 2, floppy tail indicating complete loss of tonus in tail; grade 3, floppy tail and hind limb paresis, grade 4: floppy tail and unilateral hind limb paralysis; grade 5, floppy tail and bilateral hind limb paralysis. Due to ethical reasons, mice were sacrificed if they reached grade 5 or if hind limb paralysis persisted for 2 days.

### Intrathecal injection

Mice were anesthetized using isoflurane inhalation anaesthesia (2–4% Iso-Vet, Abbott Laboratories) and received sc Temgesic (Reckitt Benckishiser Pharmaceuticals Ltd., Berkshire, UK) in isotonic sterile saline (9 mg/ml NaCl, Fresenius Kabi, Copenhagen, Denmark) for pain relief. A 30-gauge needle (bent 55° with a 2 mm tip) attached to a 50 μl Hamilton syringe was used to perform the injection into the Intrathecal space of the cisterna magna as described previously [11]. Mice received 10, 50 or 100 μg of MIS416 (Innate Immunotherapeutics, New Zealand) or MIS416-conjugated Alexa Fluor (AF) 488 [33]. Mice that received vehicle (phosphate buffered saline, PBS) were used as controls. After the injections, animals received 1 ml of isotonic sterile saline sc. The expression of IFNβ in response to intrathecal MIS416 was dose-dependent (not shown) as evaluated by in vivo imaging and was optimally induced by 100 μg, and this dose was used throughout the study.

### Tissue processing

Mice were euthanized at 2, 4 or 24 h post injection with an overdose of sodium pentobarbital (100 mg/kg, Glostrup Hospital). For histology, mice were perfused intracardially with ice-cold PBS followed by 4% paraformaldehyde (PFA). Dissected brains and spinal cords were immersed in 30% sucrose in PBS for 1 day, then frozen and 16 μ m thick tissue sections were cut on a cryostat (Leica).

For flow cytometry, mice were euthanized with an overdose of pentobarbital and then perfused intracardially with ice-cold PBS. CNS tissue was collected and held in Hank’s balanced salt solution (HBSS, Gibco, Paisley, UK).

For reverse transcriptase- quantitative polymerase chain reaction (RT-qPCR), animals were perfused with ice-cold PBS, brains and spinal cords were placed in 0,5 ml TriZol Reagent (Ambion) and stored at − 80 °C until needed for RNA extraction.

### Flow cytometry and cell sorting

A single cell suspension was obtained by digesting the tissue using Multi Tissue Dissociation Kit 1 (Miltenyi Biotec, Bergisch Gladbach, Germany) according to the manufacturer’s protocol and then forcing the dissociated CNS tissue through a 70 μm cell strainer (Falcon, USA) with HBSS supplemented with 2% fetal bovine serum (FBS, Merck, Germany) Cells were spun down and resuspended in 37% Percoll (GE Healthcare Bio-sciences AB) in a buffer made of 45 mL 10xPBS, 3 mL HCI 0.6, 132 mL water, pH 7.2 Followed by centrifugation at 2500 g for 20 min at RT. The myelin layer was removed and the cell pellet was washed. Cells were counted and incubated in blocking solution containing HBSS, 2% FBS, anti CD16/32 antibody (Clone 2.4G2, BD Biosciences, San Jose, USA) Syrian hamster IgG (50 μg/ml, Jackson ImmunoResearch Laboratories Inc., West Grove, PA, USA) and 0.01% sodium azide. The cells were then labelled with fluorophore-conjugated antibodies (BioLegend): anti-CD45 (clone 30-F11), CD11b (M1/70), F4/80 (BM8), GR-1 (RB6-8C5), Ly6G (1A8), Ly6C (HK1.4), CD11c (N418) and PDL-1 (10F.9G2), in blocking solution.

Anti-CD45.2 (104) and anti-CD45.1 (A20) antibodies (BD Biosciences) were used to distinguish respectively recipient and donor derived cells, in experiments involving chimeric mice. Fluorescence data were acquired on an LSRII flow cytometer (BD Biosciences) with FACSDiva software (BD Biosciences) and analysed with Flowlogic (Inivai Technologies). A FACS Aria III cell sorter (BD Biosciences) was used for cell sorting.

### Transfer of myeloid cells

To test the therapeutic role of CNS-infiltrating myeloid cells, healthy B6 mice or mice showing first symptoms of EAE received intrathecal MIS416 (100 μg). CNS tissues were isolated from donor mice 1 day post injection and prepared for cell sorting to obtain monocytic (CD45^hi^CD11b^hi^GR-1^low/−^F4/80^+^) and granulocytic (CD45^hi^CD11b^hi^GR-1^hi^F4/80^−^) cell populations. To test the therapeutic role of peripherally induced myeloid cells, spleens were isolated 1 day post injection from animals which received MIS416 intravenously (iv), and mechanically dissociated. Red cell lysis buffer 0.83% NH_4_CL (Merck, Germany) was used to remove erythrocytes. A pre-sort using anti-CD11b beads (Miltenyi Biotech) was performed to enrich the percentage of myeloid fraction. The positive fraction was then stained with fluorochrome-conjugated antibodies to identify neutrophils (CD11b^hi^Ly6G^+^Ly6C^dim^CD11c^−^F4/80^−^) and monocytes (CD11b^hi^Ly6G^−^Ly6C^dim/hi^CD11c^−^F4/80^low^). Selected myeloid cells were then sorted and injected intrathecally to recipient mice showing first symptoms of EAE. Mice received 1,5–2,0 × 10^5^ cells/mouse. Mice treated with sorted cells were observed over the following 4 days to assess disease progress.

### Histology

To identify the cellular source of IFNβ and localization of MIS416-AF488, frozen brain sections from YFP/IFNβ reporter mice that had received MIS416-AF488 by intrathecal injection were washed in PBS and incubated with methanol and H_2_O_2_ to saturate endogenous peroxidase. After repeated rinses with 0.2% Triton X100 in PBS (PBST) sections were incubated in blocking solution containing PBST and 3% bovine serum albumin (BSA), followed by 2 h incubation with the following primary antibodies: polyclonal rabbit anti-green fluorescent protein (GFP) (ab6556; Abcam), rabbit anti-IBA1 (Cat. No. 019–19,741 Wako), PE-conjugated rat anti-mouse CD45 (#103106, Biolegend), rat anti-mouse Ly6G (Abcam), or Cy3 conjugated monoclonal mouse anti glial fibrillary acidic protein (GFAP, Sigma-Aldrich). Sections were then washed with PBST and incubated with the appropriate secondary antibodies including biotinylated goat anti rabbit IgG (H + L) (#64256, Abcam), donkey anti-rat Alexafluor 594 (Abcam) and Alexafluor 555 donkey anti-rabbit IgG (H + L) (1:200, Life Technologies). Following incubation with secondary antibody, the sections were incubated with streptavidin–horseradish peroxidase (RPN1231V, GE Healthcare), washed in PBS and GFP staining was developed using the TSA™ System (PerkinElmer) according to the manufacturer’s instructions. Nuclei were visualized by DAPI staining and the sections were mounted with gelvatol [11]. For routine histology, sections were stained with hematoxylin and eosin (H&E) and luxol fast blue (LFB) [2, 11]. Images were acquired using an Olympus DP71 digital camera mounted on an Olympus BX51 microscope (Olympus, Ballerup, Denmark) or with an Olympus FV1000MPE Confocal and Multiphoton Laser Scanning Microscope, Danish Molecular Biomedical Imaging Center (DaMBIC), University of Southern Denmark. Images were combined using Adobe Photoshop CS3 (Adobe Systems Denmark A/S, Copenhagen, Denmark) to visualize double-labeled cells. Isotype matched control antibodies were used to test the specificity of primary antibodies as previously described [11]. This resulted in lack of staining (not shown).

### In vivo imaging

For in vivo imaging of luciferase activity, IFN-β^+/Δβ-luc^ mice were injected ip with D-luciferin (150 mg/kg), anesthetized with isoflurane and monitored using an IVIS 200 imaging system (CaliperLS) (DaMBIC). Photon flux was quantified using Living Image 4.4 software (CaliperLS).

### RNA isolation and quantitative RT-PCR (RT-qPCR)

RNA extraction was performed as described previously [11]. The extraction of RNA from sorted cells was performed using RNeasy Micro Kit (QIAGEN) according to manufacturer’s protocol. RNA concentration was measured on a NanoDrop spectrophotometer (Nanodrop® ND-1000 Spectrophotometer, Thermo Scientific, Roskilde, Denmark) and converted into cDNA using a high-capacity cDNA reverse transcription kit (Applied Biosystems). qRT-PCR was performed using an ABI Prism 7300 sequence detection system (Applied Biosystems).

The collection of primers and probe sequences listed in our previous study [11] was extended as follows: IL-6 (Forward TATGAAGTTCCTCTCTGCAAGAGA, Reverse TAGGGAAGGCCGTGGTT, Probe CCAGCATCAGTCCCAAGAAGGCAACT TAMRA), iNOS (Mm00440502, Applied Biosystems), CXCL1 (Mm04207460) and CXCL2 (Mm00436450). 18S rRNA was used for normalization of gene expression. Ct values were determined and results were expressed relative to 18S rRNA (2^ΔCT^ method). Gene expression in both brain and spinal cord was measured and results were pooled.

### RNA sequencing

RNA sequencing (RNAseq) was performed on the same RNA from above mentioned materials. In brief, RNA quality was checked using an Agilent bioanalyzer. Sequence libraries were prepared using an Illumina.Truseq Stranded mRNA sample preparation kit (Illumina, San Diego, USA) RNA sequencing was performed at the Genome Analysis Facility of the Odense University Hospital with Illumina NextSeq sequencer using 2 × 75 bp paired end reads. The raw data were first demultiplexed using CASAVA thereby creating FASTQ files. The FASTQ files were processed using BBDUK from the BBMAP tool package for trimming remnant adaptor sequences, removal of low quality bases and reads and kmer-filtering. The quality filtered reads were subsequently first aligned against the murine transcriptome (Mus_musculus. GRCm38.87), and then to the murine genome (Mus_musculus. GRCm38) using TOPHAT2. Quantification was done using the HTseq-count python module embedded in the HTSeq tool package [1]. The *edgeR* R-package [23] was used for transformation of the raw counts into counts per million (cpm), and filtration of very low expressed genes. Only genes which had cpm ≥ 1 cpm in at least 3 samples were kept for further analysis. Furthermore, the edgeR package was used for TMM normalization. Finally, the *limma* R-package was used for calculating the statistics for the differential expression analysis. KEGG pathway analysis was done using DAVID [26].

### Statistical analyses

Data were analysed by two tailed non-parametric Student’s T Test followed by Mann-Witney test, using GraphPad Prism version 6 (Graphpad Software Inc., San Diego, CA, USA). For statistical evaluation of more than two groups one-way ANOVA followed by a Bonferroni post hoc correction was used. Results are presented as mean ± SEM. Values of *p* < 0.05 were considered as significant.

## Results

### Intrathecal MIS416 induced recruitment of phagocytic monocytes and neutrophils in CNS

**Fig. 1:**
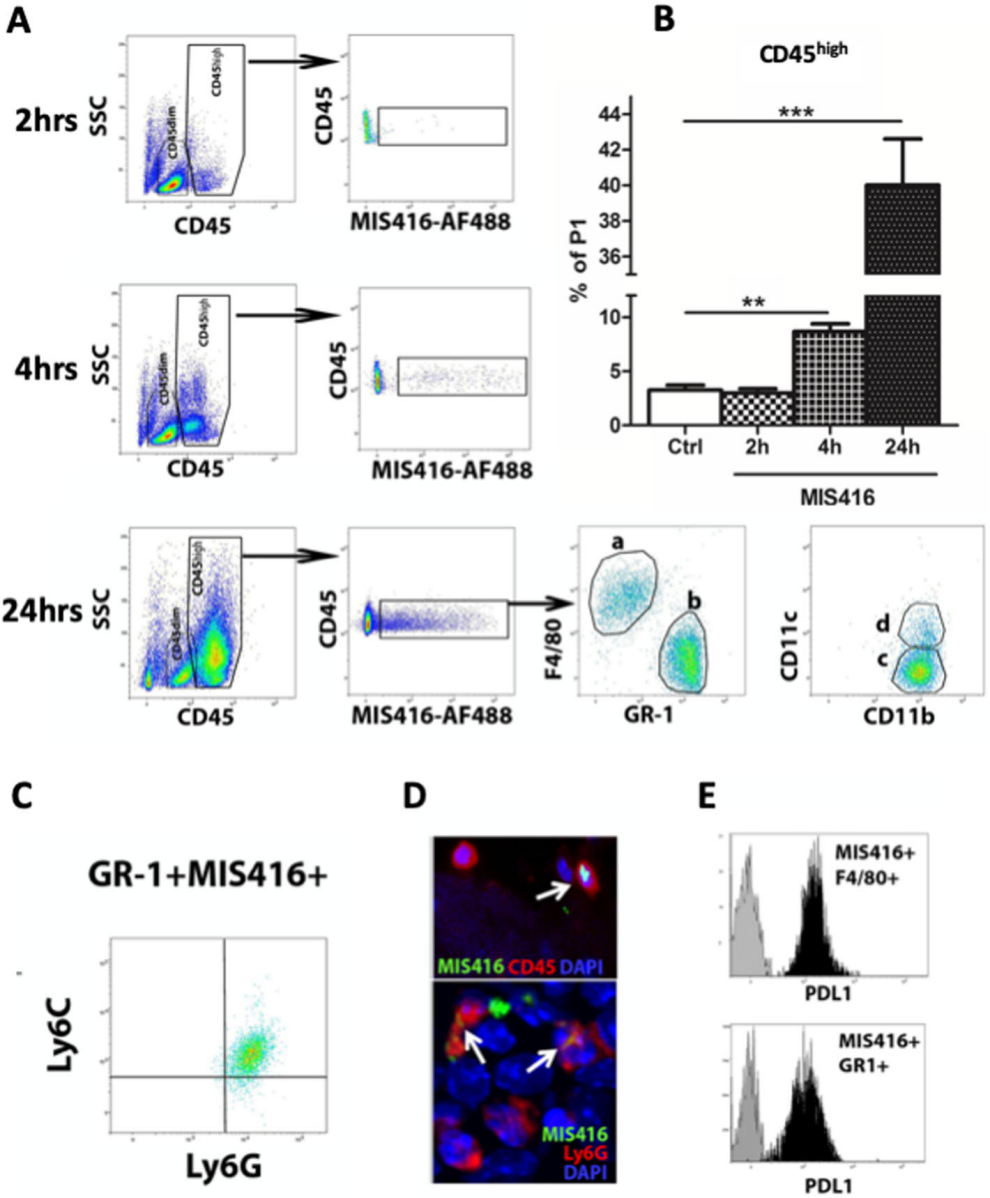
Intrathecal MIS416 is taken up by phagocytic monocytes and neutrophils in CNS. **a** Representative flow cytometry profile showing the distribution of CD45^high^ and CD45^dim^ cell populations in the CNS of control (Ctrl) and MIS416-treated mice at 2, 4 and 24 h. MIS416^+^CD45^high^ cells were detected in the CNS following intrathecal MIS416 delivery. The MIS416^+^CD11b^high^CD45^high^ cell population included F4/80^+^GR-1^low^CD11c^−/+^ (a and c and d) and F4/80^−^Gr-1^high^CD11c^−^ cells (**b** and **c**). **b** bar graphs show proportion of CD45^high^ cells at 2, 4 and 24 h. **c** Representative flow cytometry profile showing GR-1^+^MIS416^+^ cells to be Ly6c^low^Ly6G^high^. **d** Micrographs showing colocalization of MIS416 (green) with CD45^+^ or Ly6G^+^ (red) cells (arrows) located in the extraparenchymal compartment of the CNS that had received MIS416-AF88 by intrathecal injection 4 h before. DAPI (blue) shows nuclear staining. **e** MIS416^+^GR-1^high^ and MIS416^+^F4/80^+^ cells were analyzed for the expression of PDL-1 by flow cytometry. Gray histograms represent fluorescence minus one (FMO) staining, and black histograms represent positive staining for PDL-1. Data are presented as mean ± SEM. (*n* = 5–12 per group). Results were analyzed using the two-tailed Mann-Whitney u-test; * *p* < 0.05, ** *p* < 0.01, *** *p* < 0.001

The MIS416 microparticle expresses innate ligands and is designed to be phagocytosed. To test whether innate immune targeting within the CNS impacts traffic of myeloid cells to the CNS, MIS416 was administered to CSF via intrathecal injection. We observed increased numbers of CD45^high^ cells, a large proportion of which had phagocytosed MIS416 (Fig. 1a). These were detectable as early as 2 h after MIS416 administration and greatly increased by 4 h and 24 h (Fig. 1a, b). The CD45^high^ population included CD11b^+^Gr1^−^F4/80^+^ monocytic myeloid cells (Fig. 1a) as well as CD4^+^TCRβ^+^ T cells (not shown). A proportion of MIS416-phagocytosing CD11b^+^F4/80^+^Gr1low/^−^ cells (30%) also expressed CD11c (Fig. 1a). A significant proportion (30%) of cells that had phagocytosed MIS416 after intrathecal injection were CD11b^+^Gr1^high^F4/80^−^ granulocytes, confirmed to be neutrophils by flow cytometric staining for Ly6C^low^Ly6G^high^ (Fig. 1c) and by immunofluorescent microscopy and nuclear morphology (Fig. 1d). Fluorescence microscopy confirmed that intrathecally-administered fluorescent MIS416 was taken up by extraparenchymal CD45^+^ including Ly6G^+^ cells (Fig. 1d). Both MIS416^+^Gr1^high^ and MIS416^+^F4/80^+^ cell populations expressed PDL-1 (Fig. 1e).

### MIS416-phagocytosing cells within the CNS are recruited from blood

**Fig. 2:**
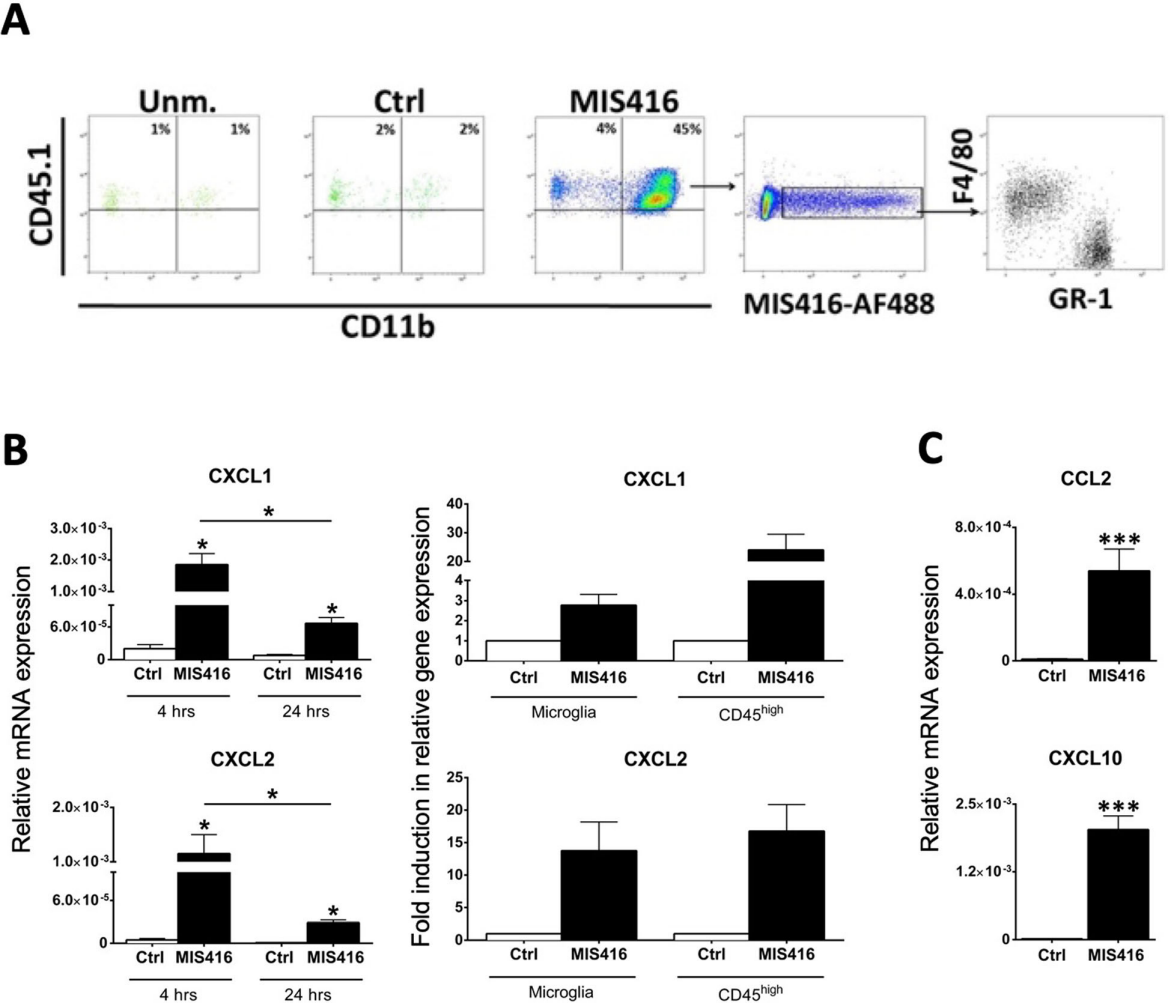
MIS416 treatment induced recruitment of blood-derived myeloid cells to the CNS and induced chemokine expression by CNS myeloid cells. **a** CD45.2 mice were irradiated and received bone marrow transplant from CD45.1 donors, and 8 weeks later mice received MIS416 intrathecally and 24 h post injection brains were isolated and analyzed. Representative flow cytometry profile showing the distribution of CD45.1^high^ cell population in the CNS of unmanipulated (Unm), control (Ctrl) and MIS416-treated CD45.2 recipient mice at 24 h. **b** Expression of CXCL1 and CXCL2 in the CNS from MIS416 treated or control mice at 4 and 24 h was analyzed by RT-qPCR. Bar graphs show CXCL1 and CXCL2 gene expression at indicated times. **b** Fold changes in CXCL1 and CXCL2 mRNA in sorted CD45^dim^CD11b + microglia and CD45^high^ cells from MIS416-treated mice compared to control mice, analyzed by RT-qPCR. **c** Expression of CCL2 and CXCL10 mRNA in the CNS from MIS416-treated or control mice at 4 h was analyzed by RT-qPCR. Bar graphs show CCL2 and CXCL10 gene expression. Data are presented as means ± SEM. (*n* = 3–5 per group). Results were analyzed using the two-tailed Mann-Whitney u-test; * *p* < 0.05, ** *p* < 0.01, *** *p* < 0.001

To verify that phagocytic monocytes and neutrophils in the CNS originated from blood, we used bone marrow chimeric mice, exploiting CD45 allotype differences. CD45.2 mice were head-shield irradiated and received CD45.1 bone marrow. Mice received intrathecal MIS416 and the composition of MIS416-phagocytosing cells was analyzed at 24 h post injection. A large proportion of MIS416-phagocytosing CD45^high^ cells in the brains of chimeric mice expressed the donor CD45.1 allotype (Fig. 2a) indicating that they had infiltrated from blood. Blood-derived MIS416-phagocytosing cells included both monocytic myeloid cells (F4/80^+^GR-1^low/−^) and neutrophils (F4/80^−^Gr-1^high^), in similar proportions to those seen in non-chimeric mice (Fig. 1a). These findings suggested that intrathecal MIS416 induced a CNS-intrinsic inflammatory program, which led to recruitment from blood of monocytes and neutrophils into the CNS. Levels of message for both CXCL1 and CXCL2, neutrophil-recruiting chemokines were measured (Fig. 2) by qPCR. Both chemokines were significantly increased, their expression levels at 4 h being significantly higher than those at 24 h (Fig. 2b). Cell sorting showed that intrathecal MIS416 induced CXCL1 and CXCL2 mRNA in both microglia and CD45^high^ cells (Fig. 2b). Likewise, message for CCL2 and CXCL10, involved in monocyte recruitment, were significantly increased in brains of mice treated with MIS416 (Fig. 2c).

### Intrathecal MIS416-induced neutrophils inhibited progression of EAE

**Fig. 3:**
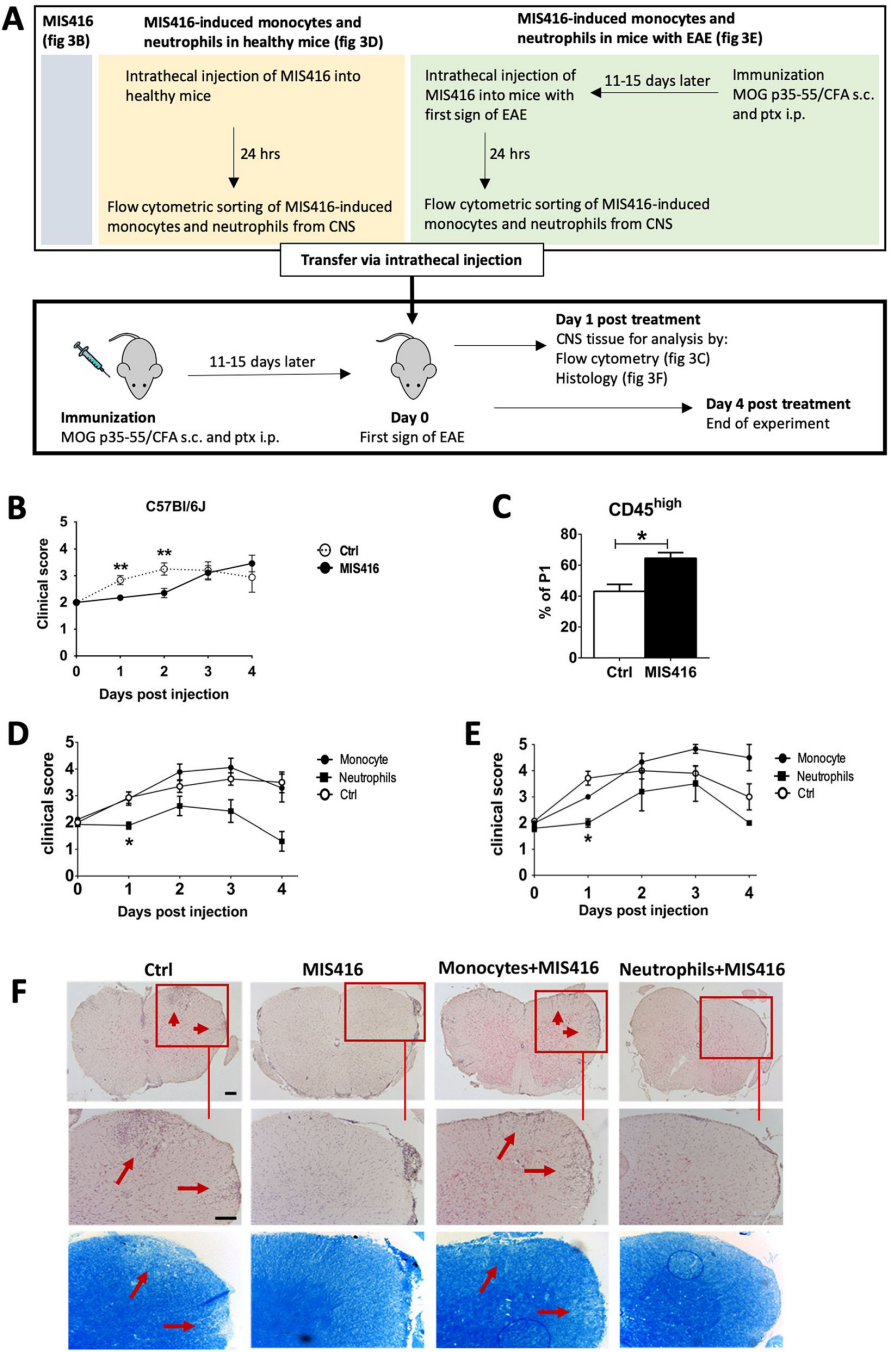
Intrathecal MIS416-induced neutrophils inhibited progression of EAE. Mice were immunized with MOG p35–55 to induce EAE and clinical signs were scored daily. **a** Flowchart showing the experimental design. **b** Clinical scores of mice with EAE. Shown is pooled data from three individual experiments. Mice treated by intrathecal administration of MIS416 (*n* = 17), at EAE onset, progressed less rapidly following 2 days than control animals(*n* = 12). **c** Cells isolated from brains of mice with EAE were analyzed by flow cytometry, at 24 h post intrathecal administration of MIS416 (*n* = 8–19). Bar graph shows MIS416-induced recruitment of CD45^high^ cells in the CNS. **d**, **e** Monocytes or neutrophils were sorted from the CNS of normal mice or mice with EAE 1 day after they received intrathecal MIS416. Cells were transferred to mice on the day of first onset of EAE. **d** Clinical score of disease progression in mice that received intrathecal injection of neutrophils and monocytes that were sorted from normal mice (*n* = 9–14 per group). **e** Clinical scores of mice that received intrathecal injection of neutrophils and monocytes sorted from donor mice with EAE (*n* = 3–7 per group), that had received intrathecal MIS416 at the onset of disease, 24 h previously. **f** Images show H&E - and LFB staining of spinal cord sections from mice with EAE, 1 day after receiving either intrathecal MIS416, Neutrophils and monocytes induced by MIS416. Red boxes show higher magnification of selected areas of spinal cord sections stained with H&E staining (arrows point at parenchymal infiltrates) and corresponding areas with LFB staining (arrows point at areas with loss of LFB). Scale bars 100 μm. Data are presented as mean ± SEM. Results were analyzed using the two-tailed Mann-Whitney u-test; * *p* < 0.05, ** *p* < 0.01, *** *p* < 0.001

B6 mice were immunized with MOG 35–55 and at day of EAE onset, defined as loss of tail tonus, they were randomized and administered MIS416 or PBS into the CSF via cisterna magna. Figure 3a shows a flowchart explaining the experimental design. Mice were then evaluated for clinical symptoms over the following 4 days. Figure 3b shows pooled data from three independent experiments.

Mice treated with a single intrathecal injection of MIS416 showed marked attenuation of disease progression compared to control animals. Clinical scores in PBS-treated mice increased at 24 h and 48 h, but did not change in mice receiving intrathecal MIS416, until the 48 h time-point, after which there was no difference between groups (Fig. 3b). Flow cytometry analysis showed that intrathecal MIS416 enhanced recruitment to the CNS of CD45^high^ cells, over levels already due to EAE (Fig. 3c).

We then asked which of the myeloid lineages recruited to the CNS by MIS416 were responsible for EAE suppression. Mice at day of first onset of EAE received monocytes or neutrophils sorted from the CNS of mice that had received intrathecal MIS416 24 h earlier (Fig. 3d and e). Only mice that received intrathecal injection of neutrophils showed attenuation of disease progression (Fig. 3d). Similar results were achieved when we transferred monocytes and neutrophils that were sorted from mice with EAE, that had received intrathecal MIS416 24 h previously (Fig. 3e). Thus CNS-infiltrating neutrophils that were recruited and activated by intrathecal MIS416 exert a suppressive function in EAE.

Histological analysis of spinal cords from EAE mice showed reduced parenchymal infiltration (H&E staining) and reduced loss of LFB staining in mice treated with MIS416 or that received neutrophils induced by MIS416, compared to untreated controls and recipients of monocytes induced by MIS416 (Fig. 3f).

#### Intrathecal MIS416 induced IFNβ and inhibited EAE in an IFNAR-dependent manner

**Fig. 4:**
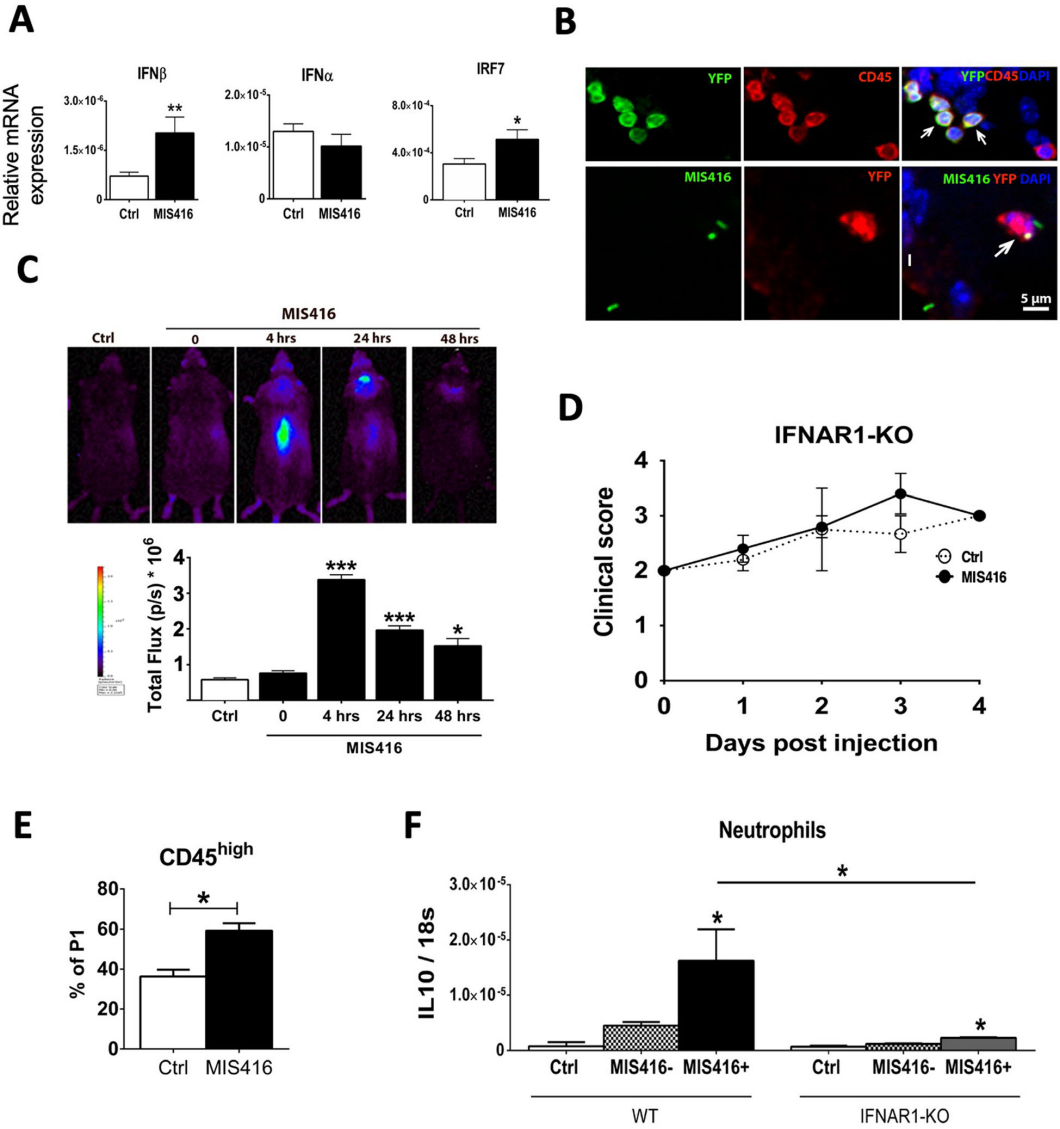
Intrathecal MIS416 transiently induced IFNβ in CNS, and inhibited progression of EAE in an IFNAR1-dependent manner. **a** Expression of IFNβ, IFNα, and IRF7 in the CNS from MIS416 treated or control mice were analyzed by RT-qPCR (*n* = 3–5 per group). **b** Micrographs showing colocalization of IFN-β/YFP+ (green) with CD45+ (red) cells (arrows) located in the extraparenchymal compartment of the CNS that had received MIS416-AF88 by intrathecal injection 4 h earlier. **b** Micrographs showing colocalization of MIS416-AF488 (green) and IFNβ/YFP (red) in a parenchymal cell (arrow). DAPI (blue) shows nuclear staining. **c** IFNβ/Luciferase reporter mice received MIS416 by intrathecal injection and luciferase activity was visualized and quantified after 4, 24 and 48 h. **c** MIS416 induced IFNβ in the brain and spinal cord. The expression was strongest at 4 h and was reduced in following days. **c** Bar graph shows the quantification of luciferase activity at indicated time points (*n* = 3–4 per group). **d** IFNAR1 deficient mice were immunized with MOG p35–55 to induce EAE and clinical signs were scored daily. The protective effect of MIS416 was not seen in mice lacking the receptor for Type I IFN (*n* = 5 per group). **e** Bar graph shows the percentage of CD45^high^ cells in the CNS of IFNAR1-deficient mice during EAE, analyzed by flow cytometry. MIS416 induced recruitment of CD45^high^ cells in the CNS (*n* = 6–9 per group). **f** Neutrophils were sorted from the brain of B6 (*n* = 6) and IFNAR1 deficient mice (*n* = 4) with EAE, 1 day post MIS416 injection, and IL-10 gene expression was analyzed by RT-qPCR. Data are presented as mean ± SEM (*n* = 3–4 per group); Results were analyzed using one-way ANOVA with Bonferroni multiple comparison test. * *p* < 0.05, ** *p* < 0.01, *** *p* < 0.001

RNAseq analysis (Additional files 1 and 2) pointed to involvement of Type I IFN in the MIS416-mediated response. Because we have shown Type I IFN to play a protective role in EAE, when induced in the CNS [11], we examined the induction of IFNβ, IFNα and IRF7 in the CNS, by RT-qPCR. Intrathecal MIS416 induced IFNβ and IRF7, but not 4 members of the IFNα family (Fig. 4a).

To examine the source of IFNβ, MIS416 was injected to IFNβ/YFP reporter mice and IFNβ expression in CNS was assessed by fluorescence microscopy for GFP. A single injection of MIS416 induced IFNβ expression in CD45^+^ cells in leptomeninges in the brain (Fig. 4b) and spinal cord (not shown). Moreover, we showed that cells which had phagocytosed MIS416-AF488 also expressed IFNβ (Fig. 4b).

The spatiotemporal pattern of the IFNβ response was examined in IFNβ-luciferase reporter mice. In vivo imaging showed that intrathecal MIS416 transiently induced expression of IFNβ in the brain and spinal cord as early as 4 h post injection (Fig. 4c). Production of IFNβ was confined to the CNS and did not involve other tissues, in the time frame of this analysis (Fig. 4c). Expression of IFNβ (luciferase total flux) was highest at 4 h (Fig. 4c) and still significantly upregulated after 24 and 48 h post-intrathecal injection of MIS416.

The protective effect of intrathecal MIS416 treatment was completely abrogated in mice that lacked IFNAR1. The progression of EAE in IFNAR1-KO mice was unaffected by MIS416 treatment (Fig. 4d). Flow cytometry analysis showed MIS416 induced recruitment of CD45^high^ cells in the CNS of IFNAR1-deficient mice during EAE (Fig. 4e), which exhibited similar phenotypes to those seen in WT mice (Fig. 1 and not shown).

Thus, lack of IFN response in MIS416-induced neutrophils abolished their capacity to inhibit EAE. Expression of IL-10 mRNA by MIS416-phagocytosing neutrophils sorted from mice with EAE at 24 h post injection, was dramatically reduced in cells from IFNAR1-deficient mice, although a significant MIS416-induced increase over baseline was still detectable (Fig. 4f).

### Selective infiltration of phagocytic monocytes to the CNS following intravenous administration of MIS416

**Fig. 5:**
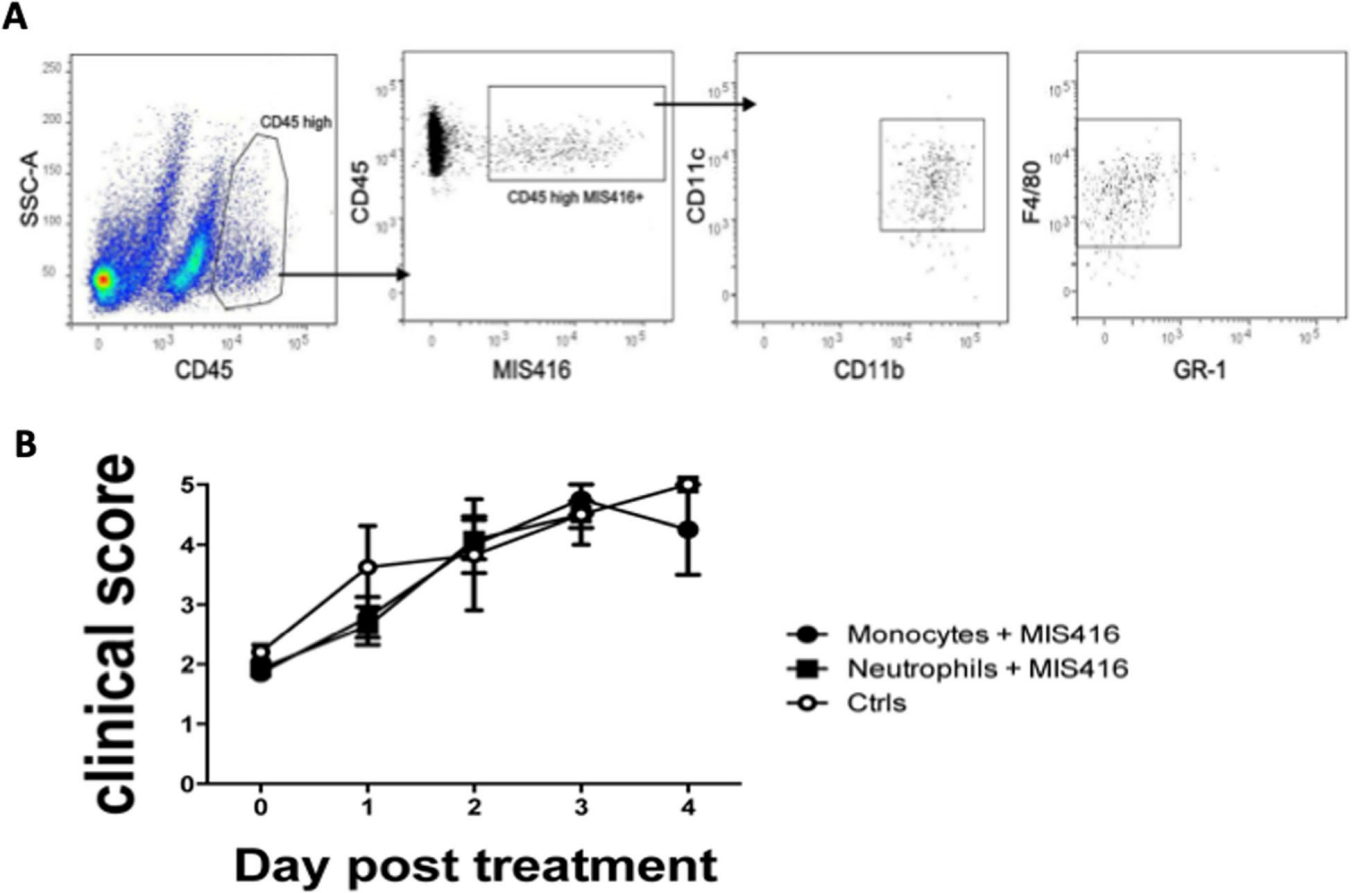
MIS416-induced myeloid cells in CNS and spleen after intravenous injection of MIS416. **a** Single cell suspensions of perfused brains were analyzed by flow cytometry, 24 h post intravenous administration of MIS416-AF488, the infiltrating myeloid cells were distinguished from microglia by differential CD45 expression. Representative flow cytometry profiles showing CD45^High^CD11b^High^MIS416^+^ cells as F4/80^+^Gr-1^−^CD11C^+^ monocytes. **b** Monocytes or neutrophils, sorted from spleens of mice that had received intravenous MIS416 24 h earlier, were transferred intrathecally to mice at the onset of EAE. The graph shows the clinical scores of mice following the 4 days post treatment (*n* = 5–7 per group)

It was previously shown that iv administration of MIS416 increased numbers of T cells, macrophages and monocytic myeloid cells, but not neutrophils, in the CNS of healthy mice, although iv MIS416 did induce an increase in splenic neutrophils [34]. We have repeated this analysis using fluorescent-tagged MIS416-AF488 and show a great predominance of monocytic myeloid CD11b^+^F4/80^+^Gr1^−^CD11c^+^ cells among the CNS-infiltrating MIS416-phagocytosing CD45^high^ cells 24 h after intravenous injection (Fig. 5a). Peripheral MIS416 did not induce expression of neutrophil-attracting chemokines in the CNS (not shown), indicating that intrathecal administration of MIS416 was required for activation of CNS intrinsic pathways that induce recruitment of neutrophils to the brain.

Neither monocytes nor neutrophils isolated from spleens of mice that had received intravenous MIS416 24 h earlier ameliorated symptoms of disease when transferred to mice at the onset of EAE (Fig. 5b). Taken together, these data indicate a necessity for signaling within the CNS to recruit EAE-suppressive neutrophils.

## Discussion

This study has addressed the role of phagocytosis within the CNS in recruitment of protective myeloid cells to the CNS. Our findings show that introduction of a bispecific TLR9 and NOD2 microparticle MIS416 to the CSF induced a local chemokine response that led rapidly to infiltration from blood of phagocytic neutrophils, which were shown by intrathecal transfers to be directly responsible for amelioration of EAE. Co-recruited phagocytic monocytes were ineffective in transfer of EAE suppression. EAE suppression by neutrophils was dependent on Type I IFN signaling which also amplified cytokine responses by the same cells. By contrast, intravenous administration of MIS416 induced monocyte but not neutrophil infiltration to the CNS, and intrathecal transfer of either cell type from spleen showed no protection against EAE.

The rapidity of the response and the CSF placement of the inducing particle point to meningeal and/or perivascular macrophages as phagocytic cell(s) that likely initiated the monocytes- and neutrophil-recruiting chemokine response. Recipient-allotype CD45^high^ cells that had phagocytosed MIS416 in BM chimeric mice included monocytic myeloid cells (F4/80^+^GR-1^low/−^) (not shown). Although some of these likely originated from blood, due to incomplete chimerism in head-shielded irradiated mice, such cells must also include CNS-resident macrophages, in which the neutrophil-recruiting chemokine response was initiated, consistent with RT-qPCR analysis of sorted cells. That analysis showed that microglia were also a source of chemokines, and indeed host allotype MIS416-phagocytosing CD45^low^ cells were also seen in BM chimeras (not shown). Consideration of kinetics of response and proximity of intrathecally-injected particles to CSF macrophages argues for the latter as key inducers of protective recruitment.

Neutrophils recruited from blood also phagocytosed MIS416. Transfer of intrathecal MIS416-induced neutrophils, but not monocytes, suppressed EAE. The collaborative activity of the innate TLR9 and NOD2 signaling pathways was therefore responsible for induction of a protective neutrophil phenotype. Such bispecific signaling, e.g. in response to tissue damage, may be important to balance pro and anti-inflammatory responses in the CNS, and be critical to restore homeostasis in the inflamed CNS. Intracellular TLR9 and NOD2 signaling led to production of IFNβ and IL-10, the level of the latter response being controlled by signaling through the Type I IFN receptor IFNAR. Whether or not members of the IFNα family other than the 4 detected by PCR were also induced, IFNAR signaling was required for EAE suppression. This is consistent with our previous studies of CNS-endogenous innate signaled EAE protection. Mechanism of EAE suppression likely included IFNAR and IL-10 signaling. A previous study showed that IL-10-expressing neutrophils isolated from the CNS of mice with EAE could suppress inflammatory T cell responses in vitro, and this was dependent on iNOS as well as IFNγ production by the T cells [35]. These were upregulated in CNS by intrathecal MIS416. Intrathecal and intravenous MIS416 both induced PDL-1+ myeloid populations, shown to be IFNγ-dependent in the case of peripheral suppression, where PDL-1+ monocytes also infiltrated the CNS [34]. PD1 inhibitory signaling may be considered as a possible mechanism for intra-CNS EAE suppression, although in the present study, PDL-1 was expressed by both the neutrophils and monocytes that were recruited by MIS416, so did not correlate with suppressive function. Suppression of EAE by iv-administered MIS416 was dependent on innate IFNγ [34], whereas we show dependence on Type I IFN. In another study we showed that IFNγ could substitute for Type I IFN signaling [2], and it cannot be excluded that an analogous functional overlap contributed in the myeloid suppression of CNS inflammation that we describe here.

There is a great interest in understanding the role of myeloid cells that traffic between peripheral tissues and CNS and how harnessing them might be a useful tool to control CNS pathologies [25]. Such cells can traffic to the brain by multiple routes (reviewed in [7]). Recruitment of myeloid cells into the CNS is normally induced and directed by chemokines [21]. Our RNAseq and RT-qPCR data showed upregulation of several chemokines and chemokine receptors in the CNS in response to intrathecal MIS416. These included chemokines that are associated with neutrophil recruitment, and KEGG pathway analysis showed a significant involvement of chemokine signaling pathways. In addition, MMPs were also among the upregulated genes. MMPs facilitate cellular infiltration of the CNS [29, 30]. These findings together indicate that intrathecal MIS416 induced recruitment of blood-derived myeloid cells via induction of chemokines and MMPs, initially by CNS-resident myeloid cells. Neutrophils have been described to be pro-pathologic and protective in different studies [12, 18, 31, 35]. This highlights the importance of understanding how to direct the relevant activity of neutrophils.

Neutrophils were not induced to infiltrate to the CNS by iv-administered MIS416. The absence of neutrophils is likely due to the lack of MIS416-induced neutrophil-recruiting chemokines in the CNS. This indicates that CNS intrinsic signaling activity is necessary to recruit suppressive neutrophils to the CNS. This reinforces that triggering of a neutrophil-attractant chemokine response within the CNS itself was a key event, and that this depended on local interactions. Previous description of EAE-protection induced by intravenous MIS416 showed mobilization of neutrophils and monocytes, increased number of splenic neutrophils, and IFNγ-dependent suppression of encephalitogenic T cell responses, but we show here that only MIS416-phagocytosing monocytes trafficked to the CNS from the periphery. Our transfer experiments would suggest that these were not suppressive, and that peripheral anti-inflammatory programs were more important in those studies [34]. We have taken advantage of MIS416 as a tool to investigate CNS endogenous signals and phagocytosis in EAE, to probe the immunological pathways that favor regulatory myeloid activity with focus on innate interferon signaling. Although MIS416 has shown promise in various studies [10, 14, 16, 32], clinical application was not our goal.

Our findings suggest major involvement of IFN associated programs in the CNS upon intrathecal challenge with MIS416. RNAseq and RT-qPCR analysis showed a significant upregulation of many IFN-I stimulated genes, including CXCL10, IRF7, MX1, OAS1 and ISG15, as well as involvement of JaK-STAT signaling pathway. The IFNAR-dependence of EAE suppression is consistent with these observations. The phagocytic capacity of CNS myeloid cells is enhanced by IFNβ that can be produced by CD45/CD11b cells in the meninges [6, 11, 13, 28]. Phagocytosis of myelin and axonal debris as well as apoptotic cells by myeloid cells is critical for maintaining CNS homeostasis [27]. Our immunofluorescence analysis in IFNβ/YFP reporter mice identified CD45+ leptomeningeal myeloid cells as cell sources of IFNβ. The induction of IFNβ within CNS in response to MIS416 may have enhanced phagocytosis, as well as activating anti-inflammatory mechanisms [4]. Increased phagocytic activity was supported by RNAseq data from MIS416-treated CNS, which showed upregulated levels of phagocytosis related genes, including MSR1 and TREM1. These are known to be expressed by monocytes and act as positive regulators of phagocytosis in these cells [8, 15]. Extraparenchymal myeloid cells would be expected to take up MIS416 microparticles directly upon encounter in leptomeningeal space.

## Conclusion

Our findings increase understanding of the effect of CNS-endogenous innate signaling to myeloid cells and how it can induce anti-inflammatory programs that can suppress MS like disease.

## Supplementary information

**Additional file 1. **Intrathecal MIS416 influences the CNS inflammatory programs. A) KEGG pathway analysis. KEGG pathway enrichment analysis of upregulated gene in mice received MIS416 by intrathecal injection identified 50 pathways (after Benjamini-correction), 6 of which are shown on the graph. Benjamini-corrected *p* values are indicated on the bar graph for each pathway. B) Expression of IFNγ, IL6, iNOS and IL-10 in the CNS from MIS416 treated or control mice were analyzed by RT-qPCR. Data are presented as mean ± SEM. (*n* = 3–5 per group). Results were analyzed using the two-tailed Mann-Whitney u-test; *** *p* < 0.001. ND; not detected.

**Additional file 2.** Intrathecal MIS416 alters CNS inflammatory programs and induces type I IFN associated signaling.

## References

[1] Anders S, Pyl PT, Huber W (2015). HTSeq--a Python framework to work with high-throughput sequencing data. Bioinformatics.

[2] Berg CT, Khorooshi R, Asgari N, Owens T (2017). Influence of type I IFN signaling on anti-MOG antibody-mediated demyelination. J Neuroinflammation.

[3] Boros P, Ochando J, Zeher M (2016). Myeloid derived suppressor cells and autoimmunity. Hum Immunol.

[4] Clausen BH, Lambertsen KL, Dagnaes-Hansen F, Babcock AA, von Linstow CU, Meldgaard M, Kristensen BW, Deierborg T, Finsen B (2016). Cell therapy centered on IL-1Ra is neuroprotective in experimental stroke. Acta Neuropathol.

[5] Girvan RC, Knight DA, O'Loughlin CJ, Hayman CM, Hermans IF, Webster GA (2011). MIS416, a non-toxic microparticle adjuvant derived from Propionibacterium acnes comprising immunostimulatory muramyl dipeptide and bacterial DNA promotes cross-priming and Th1 immunity. Vaccine.

[6] Goldmann T, Blank T, Prinz M (2016). Fine-tuning of type I IFN-signaling in microglia--implications for homeostasis, CNS autoimmunity and interferonopathies. Curr Opin Neurobiol.

[7] Harrison-Brown Meredith, Liu Guo-Jun, Banati Richard (2016). Checkpoints to the Brain: Directing Myeloid Cell Migration to the Central Nervous System. International Journal of Molecular Sciences.

[8] Jiang T, Zhang YD, Gao Q, Zhou JS, Zhu XC, Lu H, Shi JQ, Tan L, Chen Q, Yu JT (2016). TREM1 facilitates microglial phagocytosis of amyloid beta. Acta Neuropathol.

[9] Kawai T, Akira S (2011). Toll-like receptors and their crosstalk with other innate receptors in infection and immunity. Immunity.

[10] Khan AN, Kolomeyevskaya N, Singel KL, Grimm MJ, Moysich KB, Daudi S, Grzankowski KS, Lele S, Ylagan L, Webster GA, Abrams SI, Odunsi K, Segal BH (2015). Targeting myeloid cells in the tumor microenvironment enhances vaccine efficacy in murine epithelial ovarian cancer. Oncotarget.

[11] Khorooshi R, Morch MT, Holm TH, Berg CT, Dieu RT, Draeby D, Issazadeh-Navikas S, Weiss S, Lienenklaus S, Owens T (2015). Induction of endogenous type I interferon within the central nervous system plays a protective role in experimental autoimmune encephalomyelitis. Acta Neuropathol.

[12] Knier B, Hiltensperger M, Sie C, Aly L, Lepennetier G, Engleitner T, Garg G, Muschaweckh A, Mitsdorffer M, Koedel U, Hochst B, Knolle P, Gunzer M, Hemmer B, Rad R, Merkler D, Korn T (2018). Myeloid-derived suppressor cells control B cell accumulation in the central nervous system during autoimmunity. Nat Immunol.

[13] Kocur M, Schneider R, Pulm AK, Bauer J, Kropp S, Gliem M, Ingwersen J, Goebels N, Alferink J, Prozorovski T, Aktas O, Scheu S (2015). IFNbeta secreted by microglia mediates clearance of myelin debris in CNS autoimmunity. Acta Neuropathol Commun.

[14] Lee BC, Shin N, Lee JY, Kang I, Kim JJ, Lee SE, Choi SW, Webster GA, Kang KS (2018). MIS416 enhances therapeutic functions of human umbilical cord blood-derived Mesenchymal stem cells against experimental colitis by modulating systemic immune milieu. Front Immunol.

[15] Li H, Hong F, Pan S, Lei L, Yan F (2016). Silencing triggering receptors expressed on myeloid Cells-1 impaired the inflammatory response to oxidized low-density lipoprotein in macrophages. Inflammation.

[16] Luckey AM, Anderson T, Silverman MH, Webster G (2015). Safety, tolerability and pharmacodynamics of a novel immunomodulator, MIS416, in patients with chronic progressive multiple sclerosis. Mult Scler J Exp Transl Clin.

[17] Mildner A, Schlevogt B, Kierdorf K, Bottcher C, Erny D, Kummer MP, Quinn M, Bruck W, Bechmann I, Heneka MT, Priller J, Prinz M (2011). Distinct and non-redundant roles of microglia and myeloid subsets in mouse models of Alzheimer's disease. J Neurosci.

[18] Papadopoulos MC, Bennett JL, Verkman AS (2014). Treatment of neuromyelitis optica: state-of-the-art and emerging therapies. Nat Rev Neurol.

[19] Prinz M, Erny D, Hagemeyer N (2017). Ontogeny and homeostasis of CNS myeloid cells. Nat Immunol.

[20] Prinz M, Jung S, Priller J (2019). Microglia biology: one century of evolving concepts. Cell.

[21] Prinz M, Priller J (2010). Tickets to the brain: role of CCR2 and CX3CR1 in myeloid cell entry in the CNS. J Neuroimmunol.

[22] Reinert LS, Harder L, Holm CK, Iversen MB, Horan KA, Dagnaes-Hansen F, Ulhoi BP, Holm TH, Mogensen TH, Owens T, Nyengaard JR, Thomsen AR, Paludan SR (2012). TLR3 deficiency renders astrocytes permissive to herpes simplex virus infection and facilitates establishment of CNS infection in mice. J Clin Invest.

[23] Robinson MD, McCarthy DJ, Smyth GK (2010). edgeR: a bioconductor package for differential expression analysis of digital gene expression data. Bioinformatics.

[24] Russo MV, McGavern DB (2015). Immune surveillance of the CNS following infection and injury. Trends Immunol.

[25] Shechter R, Schwartz M (2013). Harnessing monocyte-derived macrophages to control central nervous system pathologies: no longer 'if' but 'how'. J Pathol.

[26] Huang Da Wei, Sherman Brad T, Lempicki Richard A (2008). Systematic and integrative analysis of large gene lists using DAVID bioinformatics resources. Nature Protocols.

[27] Sierra A, Abiega O, Shahraz A, Neumann H (2013). Janus-faced microglia: beneficial and detrimental consequences of microglial phagocytosis. Front Cell Neurosci.

[28] Teige I, Treschow A, Teige A, Mattsson R, Navikas V, Leanderson T, Holmdahl R, Issazadeh-Navikas S (2003). IFN-beta gene deletion leads to augmented and chronic demyelinating experimental autoimmune encephalomyelitis. J Immunol.

[29] Toft-Hansen H, Babcock AA, Millward JM, Owens T (2007). Downregulation of membrane type-matrix metalloproteinases in the inflamed or injured central nervous system. J Neuroinflammation.

[30] Toft-Hansen H, Nuttall RK, Edwards DR, Owens T (2004). Key metalloproteinases are expressed by specific cell types in experimental autoimmune encephalomyelitis. J Immunol.

[31] Wang X, Qiu L, Li Z, Wang XY, Yi H (2018). Understanding the multifaceted role of neutrophils in Cancer and autoimmune diseases. Front Immunol.

[32] Webster GA, Sim DA, La Flamme AC, Mayo NE (2017). Evaluation of neurological changes in secondary progressive multiple sclerosis patients treated with immune modulator MIS416: results from a feasibility study. Pilot Feasibility Stud.

[33] White M, Webster G, O'Sullivan D, Stone S, La Flamme AC (2014). Targeting innate receptors with MIS416 reshapes Th responses and suppresses CNS disease in a mouse model of multiple sclerosis. PLoS One.

[34] White MPJ, Webster G, Leonard F, La Flamme AC (2018). Innate IFN-gamma ameliorates experimental autoimmune encephalomyelitis and promotes myeloid expansion and PDL-1 expression. Sci Rep.

[35] Zehntner SP, Brickman C, Bourbonniere L, Remington L, Caruso M, Owens T (2005). Neutrophils that infiltrate the central nervous system regulate T cell responses. J Immunol.

