# Supplementary figures and images for: Innate signaling within the central nervous system recruits protective neutrophils
Source: Acta Neuropathol Commun. 2020 Jan 8;8:2. doi: 10.1186/s40478-019-0876-2 (PMC6950927; doi:10.1186/s40478-019-0876-2)

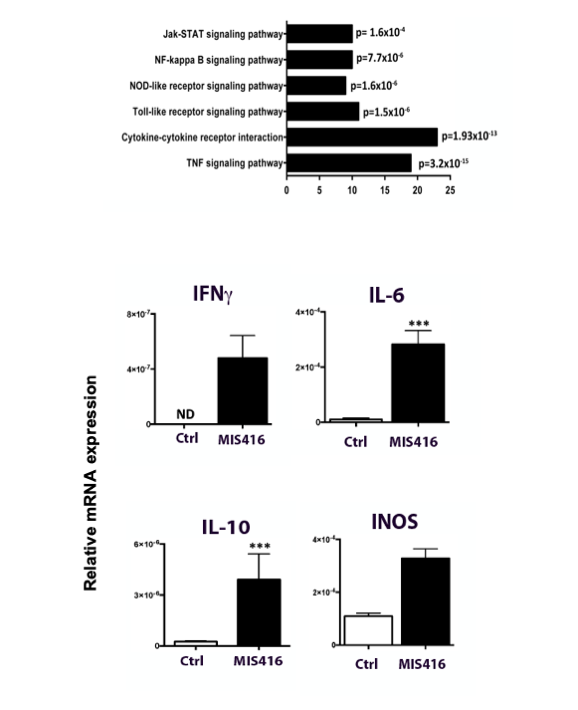

Supplement: Supplementary file 1 — Additional file 1. Intrathecal MIS416 influences the CNS inflammatory programs. A) KEGG pathway analysis. KEGG pathway enrichment analysis of upregulated gene in mice received MIS416 by intrathecal injection identified 50 pathways (after Benjamini-correction), 6 of which are shown on the graph. Benjamini-corrected p values are indicated on the bar graph for each pathway. B) Expression of IFNγ, IL6, iNOS and IL-10 in the CNS from MIS416 treated or control mice were analyzed by RT-qPCR. Data are presented as mean ± SEM. (n = 3–5 per group). Results were analyzed using the two-tailed Mann-Whitney u-test; *** p < 0.001. ND; not detected. [file 40478_2019_876_MOESM1_ESM.tiff]
