# Supplementary material for: Innate signaling within the central nervous system recruits protective neutrophils
Source: Acta Neuropathol Commun. 2020 Jan 8;8:2. doi: 10.1186/s40478-019-0876-2 (PMC6950927; doi:10.1186/s40478-019-0876-2)
Supplement: Supplementary file 2 — Additional file 2. Intrathecal MIS416 alters CNS inflammatory programs and induces type I IFN associated signaling. [file 40478_2019_876_MOESM2_ESM.docx]

**Supplementary results**

**Intrathecal MIS416 alters CNS inflammatory programs and induces type I IFN associated signaling**

To investigate the mechanism behind intrathecal MIS416 action and to assess how activation of TLR9 and NOD2 signaling by MIS416 influenced CNS inflammatory programs, we analyzed expression of inflammation-associated mediators and innate pathways by RNAseq. Intrathecal MIS416 induced significant upregulation of 164 genes in the CNS. The strongest upregulated gene was that for IL1-receptor antagonist, a known inhibitor of inflammation [4].

Notably, several chemokines, chemokine receptors and cytokines associated to neutrophil recruitment and differentiation, including CXCL1, CXCL2, CXCL3, CCL3, CCL4, Selectins (SelP, -E and -L), ICAM1, CCR1, CCR2, C5ar1, CSF3 were significantly increased in the CNS after intrathecal MIS416 treatment.

IL-6, TNF, IL-1β and matrix metalloproteinases (MMPs) 3, 8 and 13 were also upregulated. Increased IL-6 expression was confirmed by RT-PCR (Fig. Suppl).

Upregulation of macrophage scavenger receptor 1 (Msr1) and triggering receptor expressed on monocytes 1 (Trem1) indicated increased phagocytic activity.

RNA-seq analysis also showed that many Type I IFN-dependent genes were upregulated, including Mx1, Oas-2, and 3, Isg15, Socs3, Irf7, CcrI2, members of Ifi (204, 207 and 209), Ifit (1, 2 and 3), and Ifitm (3 and 6) families. Induction of IFNγ and iNOS at 4 h was shown by RT-qPCR (Fig. Suppl).

KEGG pathway analysis showed 33 chart records of which TNF signaling pathway, cytokine-cytokine receptor interaction, chemokine signaling pathway, Toll-like receptor signaling pathway, NOD-like receptor signaling pathway, NF-kappa B signaling pathway and Jak-STAT pathway were amongst the top 15 by significance (Fig. Suppl), confirming the involvement of active NOD2 and TLR9 signaling pathways.
